# Carbenoxolone and 18β‐glycyrrhetinic acid inhibit inositol 1,4,5‐trisphosphate‐mediated endothelial cell calcium signalling and depolarise mitochondria

**DOI:** 10.1111/bph.15329

**Published:** 2021-01-17

**Authors:** Charlotte Buckley, Xun Zhang, Calum Wilson, John G. McCarron

**Affiliations:** ^1^ Strathclyde Institute of Pharmacy and Biomedical Sciences University of Strathclyde Glasgow UK

**Keywords:** calcium, carbenoxolone and 18β‐glycyrrhetinic acid, endothelium, gap junctions, inositol 1,4,5‐trisphosphate (IP_3_), mitochondria, vascular

## Abstract

**Background and Purpose:**

Coordinated endothelial control of cardiovascular function is proposed to occur by endothelial cell communication via gap junctions and connexins. To study intercellular communication, the pharmacological agents carbenoxolone (CBX) and 18β‐glycyrrhetinic acid (18βGA) are used widely as connexin inhibitors and gap junction blockers.

**Experimental Approach:**

We investigated the effects of CBX and 18βGA on intercellular Ca^2+^ waves, evoked by inositol 1,4,5‐trisphosphate (IP_3_) in the endothelium of intact mesenteric resistance arteries.

**Key Results:**

Acetycholine‐evoked IP_3_‐mediated Ca^2+^ release and propagated waves were inhibited by CBX (100 μM) and 18βGA (40 μM). Unexpectedly, the Ca^2+^ signals were inhibited uniformly in all cells, suggesting that CBX and 18βGA reduced Ca^2+^ release. Localised photolysis of caged IP_3_ (cIP_3_) was used to provide precise spatiotemporal control of site of cell activation. Local cIP_3_ photolysis generated reproducible Ca^2+^ increases and Ca^2+^ waves that propagated across cells distant to the photolysis site. CBX and 18βGA each blocked Ca^2+^ waves in a time‐dependent manner by inhibiting the initiating IP_3_‐evoked Ca^2+^ release event rather than block of gap junctions. This effect was reversed on drug washout and was unaffected by small or intermediate K^+^‐channel blockers. Furthermore, CBX and 18βGA each rapidly and reversibly collapsed the mitochondrial membrane potential.

**Conclusion and Implications:**

CBX and 18βGA inhibit IP_3_‐mediated Ca^2+^ release and depolarise the mitochondrial membrane potential. These results suggest that CBX and 18βGA may block cell–cell communication by acting at sites that are unrelated to gap junctions.

**Abbreviations**
18βGA: 18β‐glycyrrhetinic acid
ΔΨ_M_
: mitochondrial membrane potential
CBX: carbenoxolone
CPA: cyclopiazonic acid
Cx: connexin
IK: intermediate conductance K^+^ channels
IP_3_
: inositol 1,4,5‐trisphosphate
PSS: physiological saline solution
SK: small conductance K^+^ channels
TMRE: tetramethylrhodamine ethyl ester

**What is already known**

- Cell–cell communication is central to endothelial control of cardiovascular function.
- Carbenoxolone and 18β‐glycyrrhetinic acid are widely used to study gap junctions in cell–cell communication.

**What does this study adds**

- Carbenoxolone and 18β‐glycyrrhetinic acid inhibit Ca^2+^ release evoked by inositol 1,4,5‐trisphosphate.
- Carbenoxolone and 18β‐glycyrrhetinic acid collapse the mitochondrial membrane potential.

**What is the clinical significance**

- Carbenoxolone and 18β‐glycyrrhetinic acid may inhibit cell–cell communication at sites unrelated to gap junctions.

## 1 INTRODUCTION

Cell–cell communication is a central component of endothelial function that is required for propagated vasodilation, transfer of signals from activated cells and emergent signalling (Bagher & Segal, 2011; Lee et al., 2018; Longden et al., 2017; McCarron et al., 2019; Socha, Domeier, Behringer, & Segal, 2012; Tallini et al., 2007). Among key signalling molecules that are transferred between cells are inositol 1,4,5‐trisphosphate (IP_3_) and cytoplasmic Ca^2+^. Changes in IP_3_ and cytoplasmic Ca^2+^ concentration decode information held in extracellular activators and encode intracellular signals that regulate the production of NO, prostacyclin and signalling peptides that diffuse to smooth muscle cells (Tran & Watanabe, 2006).

In the endothelium, Ca^2+^ increases begin as highly localised subcellular events caused by the opening of a single or multiple IP_3_ receptors in the internal store (Bagher et al., 2012; Ledoux et al., 2008; Sonkusare et al., 2012; Wilson et al., 2019). These local signals rapidly grow and propagate among cells to transmit information. However, the mechanisms that scale the signals to propagate waves and enable cell–cell communication are not well understood, even though they are critical to permit Ca^2+^ to act as a communicator with wide reach (Behringer, Socha, Polo‐Parada, & Segal, 2012; Billaud et al., 2014; Emerson & Segal, 2000a; Emerson & Segal, 2000b; Ledoux et al., 2008; Sonkusare et al., 2012; Taylor & Francis, 2014).

Several reports describe a central role for specialised intercellular connections (gap junctions) in facilitating cell–cell communication and the transmission of Ca^2+^ signals in endothelial cells (Boittin et al., 2013; Kameritsch, Pogoda, Ritter, Munzing, & Pohl, 2012). Gap junctions operate via membrane‐bound connexin hexamers that pair with connexins on adjacent cells (Bai, Yue, & Aoyama, 2018). The paired connexins form functional junctions between the membranes through which the cytoplasm of each cell may be linked (see Saez, Berthoud, Branes, Martinez, & Beyer, 2003). The connection permits intercellular movement of ions, for example, Ca^2+^, and small molecules with a mass of up to ~1.2 kDa, such as ATP (Goldberg, Moreno, & Lampe, 2002), cAMP, IP_3_ (Hernandez et al., 2007), or ROS (Billaud, Marthan, Savineau, & Guibert, 2009; Taniguchi Ishikawa et al., 2012).

Among the most widely used pharmacological agents to study the role of gap junctions in cell–cell communication are the connexin and gap junction blockers 18β‐glycyrrhetinic acid (18βGA) and its derivative carbenoxolone (CBX). Derived from the liquorice root *Glycyrrhiza glabra* , 18βGA (see Bodendiek & Raman, 2010) blocks a wide range of connexins such as Cx43 (Guan, Wilson, Schlender, & Ruch, 1996), Cx46 and Cx50 (Bruzzone, Barbe, Jakob, & Monyer, 2005). CBX is a derivative of 18βGA and is perhaps the most widely used broad‐spectrum connexin channel and gap junction inhibitor.

To investigate whether or not gap junctions play a role in endothelial IP_3_‐mediated Ca^2+^ signal propagation between cells, we aimed to disrupt normal gap junction function pharmacologically using CBX and 18βGA. IP_3_‐evoked intercellular Ca^2+^ waves were measured in the endothelium of intact mesenteric resistance arteries after stimulation with either ACh or photorelease of caged‐IP_3_ (cIP_3_). cIP_3_ provides precise spatial and temporal control of the site of cell activation and Ca^2+^ release. Paired cellular responses to ACh or cIP_3_ were analysed before and after various pharmacological interventions with CBX and 18βGA. Intercellular Ca^2+^ waves were blocked by CBX and 18βGA, but this occurred by inhibition of IP_3_‐evoked Ca^2+^ release rather than block of gap junction‐mediated signal propagation. The inhibition of IP_3_‐evoked Ca^2+^ release by CBX and 18βGA was reversible and was unaffected by the presence of small or intermediate K^+^‐channel blockers. Furthermore, CBX and 18βGA each also rapidly and reversibly collapsed the mitochondrial membrane potential. These results suggest that CBX and 18βGA act at sites outwith gap junctions by inhibiting IP_3_‐mediated Ca^2+^ release and depolarising mitochondrial membrane potential (ΔΨ_M_). Care is required in the use of these drugs when IP_3_‐mediated Ca^2+^ signalling is being investigated.

## 2 METHODS

### 2.1 Animals

All animal care and experimental protocols were carried out in accordance with the prior approval of the University of Strathclyde Animal Welfare and Ethical Review Body and under relevant UK Home Office Regulations, [Schedule 1 of the Animals (Scientific Procedures) Act 1986, UK]. Animal studies are reported in compliance with the ARRIVE guidelines (Percie du Sert et al., 2020) and with the recommendations made by the *British Journal of Pharmacology* (Lilley, Stanford et al., 2020).

Strathclyde Biological Procedures Unit is a conventional unit which undertakes FELASA quarterly health monitoring. Male Sprague–Dawley rats (10–12 week old; 250–350 g), from an in‐house colony, were used for the study. The animals were housed three per cage, and the cage type was North Kent Plastic model RC2F with nesting material “Sizzle Nest.” A 12:12 light dark cycle was used with a temperature range of 19–23°C (set point 21°C) and humidity levels between 45% and 65%. Animals had free access to fresh water and SDS diet RM1 (rodent maintenance). The enrichment in the cages was aspen wood chew sticks and hanging huts.

Animals were killed by cervical dislocation and the mesenteric bed removed. All experiments were performed using first‐ or second‐order mesenteric arteries. Controls and experimental treatments were carried out in the same tissue, so blinding and randomisation were not used.

### 2.2 Mesenteric artery preparation and mounting

Arteries were dissected, cut open longitudinally, and pinned out on Sylgard blocks using 50 μm diameter pins to expose the endothelial layer (Lee et al., 2018; Wilson, Lee, & McCarron, 2016; Wilson et al., 2019). Arteries were dissected in a physiological saline solution (PSS: 145 mM NaCl, 2 mM MOPS, 4.7 mM KCl, 1.2 mM NaH2PO4, 5 mM glucose, 0.02 mM EDTA, 1.17 mM MgCl, 2 mM CaCl, pH 7.4). PSS or a high K^+^ PSS (composition below) was used in all experiments. Endothelial cells were loaded with the Ca^2+^ indicator dye Cal‐520 (5 μM in PSS + 0.02% Pluronic F‐127, 30 min, 37°C) and then mounted in a custom flow chamber (Wilson, Lee, & McCarron, 2016).

### 2.3 Image acquisition

**FIGURE 1:**
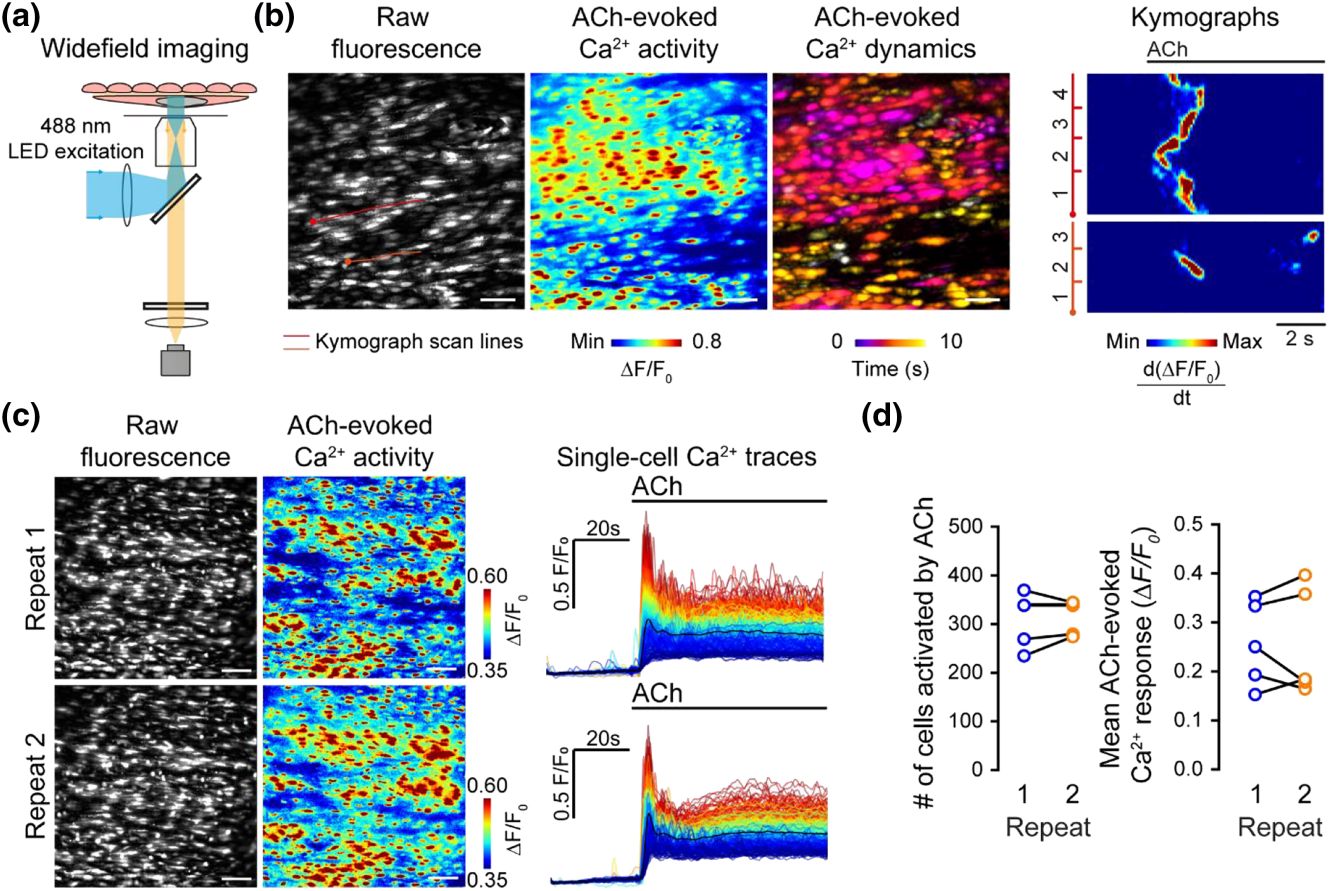
ACh‐evoked Ca^2+^ increases are reproducible. (a) Schematic of widefield microscopy for endothelial cell imaging of intact arteries. (b) Representative Ca^2+^ images and kymograph illustrating temporal dynamics of ACh (50 nM)‐evoked endothelial Ca^2+^ activity. Ca^2+^ images show raw fluorescence (left), ΔF/F_0_ maximum intensity projection (middle), and temporally colour‐coded projection of active Ca^2+^ wave fronts (determined by sequential subtraction). The kymographs show changes in Ca^2+^ levels across scanlines spanning four (red) or three (orange) cells. (c) Example of raw and pseudocoloured Ca^2+^ images and corresponding single‐cell Ca^2+^ traces (black line average) illustrating the response of a single field of endothelial cells to repeat application of ACh (50 nM, 30‐min equilibration between recordings). (d) Summary data showing no significant changes in the number of cells activated by successive ACh applications (left; 306 ± 25 cells for repeat 1, 311 ± 16 cells for repeat 2, *n* = 5) and the mean amplitude of the Ca^2+^ response (right; 0.26 ± 0.04 ΔF/F_0_ for repeat 1, 0.26 ± 0.05 ΔF/F_0_ for repeat 2; *n* = 5). All image scale bars = 50 μm

Two imaging systems were used. The first was a Nikon Eclipse TE300 inverted microscope fitted with a CoolLED pE‐300 LED illumination system (488 and 561 nm excitation) and custom designed, dual FITC/TRITC filter sets (Figure 1a). A 40× 1.3 NA Nikon S Fluor oil‐immersion objective lens was used for Ca^2+^ imaging experiments, while a 100× 1.3NA Nikon S‐Fluor lens was used in experiments imaging mitochondrial membrane potential. The second imaging system was a Nikon Eclipse FNI upright microscope equipped with a Nikon Fluor 40× 0.8 NA water immersion objective lens and a pE‐4000 CoolLED system (470 nm). This system was used for K^+^‐channel blocking experiments. All images were acquired by Andor iXon EMCCD cameras (1024 × 1024) using MicroManager v1.4.22 (Edelstein et al., 2014).

### 2.4 Localised IP_3_ uncaging

In experiments in which endothelial Ca^2+^ responses were evoked by photolysis of caged IP_3_ the endothelium was dual loaded with Cal‐520/AM and with a membrane‐permeant caged IP_3_ (cIP_3_; 5 μM) for 30 min at 37°C (Buckley, Wilson, & McCarron, 2019; McCarron, Chalmers, MacMillan, & Olson, 2010; McCarron & Olson, 2008). Photolysis of cIP_3_ was achieved using a Rapp Optoelectronics flash lamp (00‐325‐JML‐C2) at 300 V, which produced light of ~1 ms duration. The flashlamp output was passed through a 395 nm short pass filter into a 1250 μm diameter light guide (Figure 3a). The light‐guide was coupled to the epi‐illuminator of the TE300 microscope, and the output was focussed on the endothelium using broadband light. For each imaging session, broadband light was used to identify the position of the uncaging region (~70 μm diameter) and determine which endothelial cells were directly activated by the spot photolysis system.

In some experiments, the extent of IP_3_ uncaging was graded by attenuating the photolysis light power using neutral density filters placed in the excitation path. The neutral density filters had ODs of 0.5 (27% transmission at 395 nm; product code NE505B; Thor Labs, UK), 0.2 (63% transmission at 395 nm; NE502B; Thor Labs, UK), or 0.1 (80% transmission at 395 nm; NE501B; Thor Labs, UK). These experiments were performed such that the 27% transmission was recorded first, followed by 63% transmission with 15 min rest between photolysis events, and so on.

### 2.5 Experimental protocols

In experiments that examined the effect of CBX and 18βGA on IP_3_‐mediated Ca^2+^ release, ACh‐ or cIP_3_‐evoked endothelial Ca^2+^ activity was measured at 10 Hz. Baseline Ca^2+^ activity was recorded for 30 s, and then endothelial Ca^2+^ activity evoked by ACh (50 nM) or photolysis of cIP_3_. The same arteries were then incubated with CBX (100 μM, 5 min) or 18βGA (40 μM, 45 min). ACh/cIP_3_‐evoked Ca^2+^ activity was then recorded again. In separate experiments, this protocol was repeated with an additional washout period of 1 h (PSS, 1.5 ml·min^−1^) before an additional recording was taken.

In experiments assessing the effect of K^+^‐channel blockade on endothelial Ca^2+^ signalling, ACh‐evoked (50 nM) Ca^2+^ activity was assessed in the absence and then the presence of either the K_Ca_2.x channel blocker, apamin (100 nM, 10 min pre‐incubation) or the K_Ca_3.1 channel blocker, TRAM‐34 (1 μM; 10 min pre‐incubation). After their introduction, K^+^‐channel blockers remained in the PSS until washout, as indicated. In all experiments, there was a minimum of 15 min between successive stimulations for responses to recover.

Endothelial cell mitochondrial membrane potential (ΔΨ_M_) was assessed using the membrane potential‐sensitive fluorophore, tetramethylrhodamine ethyl ester (TMRE; 120 nM in PSS) (Alexander, Kelly et al. 2019; Wilson, Lee, & McCarron, 2016). Arteries were incubated in PSS containing TMRE for 10 min. Subsequently, TMRE (120 nM) was continuously present in all perfusion solutions. Images of TMRE fluorescence (excited at 555 nm) were acquired at 2 Hz for 5 min, with either CBX (100 μM in 120 nM TMRE) or 18βGA (40 μM in 120 nM TMRE) added after ~1 min baseline recording.

In a separate series of experiments, the effects of CBX and 18βGA on ΔΨ_M_ were investigated while changes in the plasma membrane potential were prevented. In these experiments, a high K^+^/Ca^2+^‐free PSS (79.7 mM NaCl, 2 mM MOPS, 70 mM KCl, 1.2 mM NaH_2_PO_4_, 5 mM glucose, 0.02 mM EDTA, 2 mM NaPy, 1 mM MgCl, 1 mM EGTA) was used to prevent plasma membrane potential changes.

In experiments where cell viability was assessed, propidium iodide (1.5 μM) was added into the PSS, 100 images were acquired and an average image intensity projection generated using Fiji (Schindelin et al., 2012). Propidium iodide was then washed out (10 min) with PSS and the experiment continued.

In experiments in which Ca^2+^ store content was assessed, the SERCA inhibitor cyclopiazonic acid (CPA; 5 μM) was applied in a Ca^2+^‐free bath solution. By inhibiting SERCA, CPA disrupts the store uptake‐leak equilibrium so that the leak may be measured as a rise in cytoplasmic Ca^2+^ concentration and integrated to determine the store content. In these experiments, CBX was used to inhibit IP_3_ receptor activity and the effectiveness of block confirmed by the absence of a response to ACh (50 nM). The bathing media was then changed to Ca^2+^‐free PSS containing CPA and the whole‐field Ca^2+^ signal profile measured over the next 15 min. The area under the Ca^2+^ discharge curve was calculated as a measure of store Ca^2+^ content and compared to controls.

### 2.6 Ca^2+^ signal analysis

Single‐cell Ca^2+^ signals were extracted from Ca^2+^ imaging data as previously described (Wilson, Lee, & McCarron, 2016). In brief, automated Fiji macros were used to extract cell coordinates and track cell positions between datasets. Single‐cell Ca^2+^ signals were then extracted and processed using a custom algorithm written in the Python programming language (Wilson, Lee, & McCarron, 2016; Wilson, Saunter, Girkin, & McCarron, 2015; Wilson, Saunter, Girkin, & McCarron, 2016). Raw fluorescence (*F*) signals were converted to baseline‐corrected fluorescence intensity (F/F_0_) by dividing each intensity measurement by the average value of a 100‐frame baseline period at the start of each trace. F/F_0_ signals were smoothed using a 21‐point third‐order polynomial Savitzky–Golay filter, and key signal parameters (e.g., amplitude, frequency, number of cells, and time of event) extracted automatically. Analysis of cIP_3_‐evoked Ca^2+^ activity was restricted to those cells in which cIP_3_ was photolysed. This was achieved by applying a mask restricted to the photolysis region. The photolysis region occupied a fraction of the overall field, so these experiments had a lower number of cells per experiment than those of ACh‐evoked signalling.

To visualise Ca^2+^ wave propagation, we created images of active Ca^2+^ wavefronts by calculating ΔF/F_0_ for each image in the recording. For cIP_3_‐evoked Ca^2+^ experiments, a maximum intensity projection of the first 3 images immediately following uncaging was taken, ensuring that only signal from the uncaging area is presented. This only differs in Figure 5, where a maximum intensity projection of the first 5 s immediately following uncaging is presented for each experimental condition to compare propagation extent. For ACh experiments, a maximum intensity projection of the 60 s after ACh onset was taken. A JET LUT was then applied to the images. Since all experiments were paired, images were contrast matched for control and treatment. To visualise mitochondria, images were loaded into FIJI and an unsharp mask applied, the background was subtracted, a Gaussian blur was applied, and the local contrast was enhanced. To get a fluorescence intensity trace, images were stabilised, and a region of interest was placed over the mitochondria of interest.

### 2.7 Data and statistical analysis

Graphical summary data represent averaged, paired responses in arteries from ≥5 different animals. Data are summarised as mean ± SEM. Data were assessed for variance homogeneity (*F*‐test) before statistical tests were performed. Raw peak F/F_0_ responses were analysed statistically using either a paired Student's *t*‐test or a paired one‐way ANOVA with Tukey's multiple comparisons test on Prism where an appropriate *F* value was achieved, version 6.0 (GraphPad, La Jolla, CA, USA). *P* < 0.05 was considered statistically significant. The data and statistical analysis comply with the recommendations of the *British Journal of Pharmacology* on experimental design and analysis in pharmacology (Curtis et al., 2018).

### 2.8 Materials

Caged IP_3_ was obtained from SiChem (Bremen, Germany). Cal‐520/AM and TMRE were obtained from Abcam (Cambridge, MA, USA). Pluronic F‐127 was obtained from Invitrogen (Carlsbad, CA, USA). TRAM‐34 and apamin were obtained from Tocris (Bristol, UK). CBX and 18βGA, ionomycin, ACh, propidium iodide and all other chemicals were obtained from Sigma (St Louis, MO, USA). All solutions were freshly prepared each day.

### 2.9 Nomenclature of targets and ligands

Key protein targets and ligands in this article are hyperlinked to corresponding entries in the IUPHAR/BPS Guide to PHARMACOLOGY (http://www.guidetopharmacology.org) and are permanently archived in the Concise Guide to PHARMACOLOGY 2019/20 (Alexander, Cidlowski et al., 2019; Alexander, Fabbro et al., 2019; Alexander, Kelly et al., 2019; Alexander, Mathie et al., 2019).

## 3 RESULTS

In the endothelium, muscarinic receptor stimulation, using the physiological agonist ACh (50 nM), evoked heterogeneous increases in Ca^2+^. The Ca^2+^ rise propagated regeneratively, initially within and subsequently between cells, to generate multicellular Ca^2+^ waves (Figure 1a,b, Video S1). These Ca^2+^ waves are the result of IP_3_‐dependent Ca^2+^ release from intracellular stores (Buckley et al., 2019; Wilson, Lee, & McCarron, 2016). In control experiments, repeated application of ACh evoked reproducible increases in Ca^2+^ and propagating waves (Figure 1c,d). There was no difference in the number of cells or the amplitude of responses on each activation with ACh.

**FIGURE 2:**
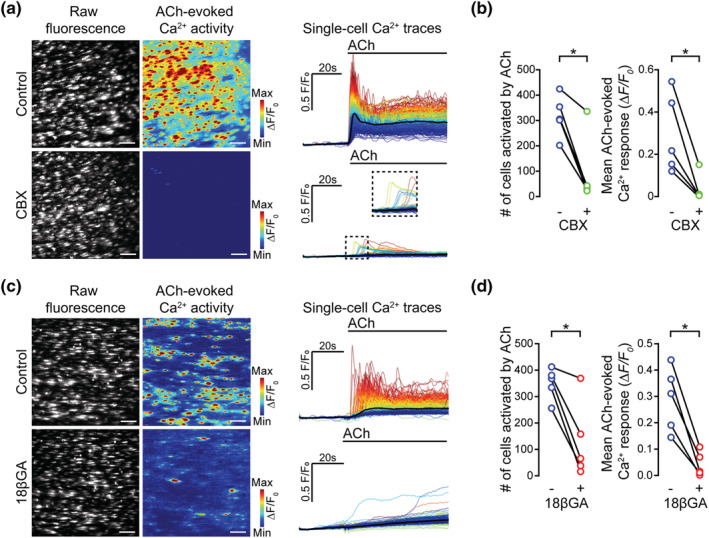
Putative gap junction blockers inhibit ACh‐evoked endothelial Ca^2+^ responses. (a–d) Effect of carbenoxolone (a, b; CBX, 100 μM, 5 min incubation) and 18βGA (c, d; 40 μM, 45 min incubation) on ACh‐evoked (50 nM) endothelial cell Ca^2+^ signalling. Panels (a) and (c) show raw baseline Ca^2+^ images, ACh‐evoked Ca^2+^ activity images (pseudocoloured max ΔF/F_0_), and corresponding single‐cell Ca^2+^ traces (black line average) obtained from the same field of endothelial cells before and after incubation with the indicated inhibitor; (b, d) (left panels) paired summary data plots showing significant decrease in the number of cells activated by ACh, for CBX (316 ± 36 cells for control, 93 ± 60 cells for CBX; *n* = 5) and for 18βGA (349 ± 27 cells for control, 129 ± 64 cells for 18βGA; *n* = 5); panels (b) and (d) (right panels) show the mean amplitude of the ACh‐evoked Ca^2+^ response before and after CBX (0.29 ± 0.08 ΔF/F_0_ for control, 0.04 ± 0.03 ΔF/F_0_ for CBX; *n* = 5) and 18βGA (0.29 ± 0.05 ΔF/F_0_ for control, 0.04 ± 0.02 ΔF/F_0_ for 18βGA; *n* = 5). * *P*<0.05, significantly different as indicated; paired *t*‐test. All image scale bars = 50 μm

It is unclear how these waves are transmitted between neighbouring endothelial cells. A prime candidate for the transmission is the movement of small molecules such as Ca^2+^ or IP_3_ through gap junctions between endothelial cells (Pohl, 2020). To explore the role of gap junctions in the intercellular propagation of Ca^2+^ waves, we examined the effects of the two widely used putative gap junction blockers, CBX and 18βGA, on ACh‐evoked endothelial cell Ca^2+^ signalling (Figure 2). The expectation in these experiments was that the drugs would reduce transmission of signals, without altering the initial Ca^2+^ increase in cells directly activated by ACh.

CBX (100 μM) and 18βGA (40 μM) each significantly reduced the number of cells responding to ACh and the amplitude of ACh‐evoked responses (Figure 2b,d).

These results initially appeared to be consistent with a contribution of gap junctions to the propagation of endothelial cell Ca^2+^ waves. However, the decrease in amplitude of ACh‐evoked Ca^2+^ signals occurred approximately uniformly across all endothelial cells—an unexpected finding, as these drugs would not be expected to reduce Ca^2+^ signals in cells directly activated by ACh. These results raised the possibility that CBX and 18βGA may each directly inhibit IP_3_‐evoked Ca^2+^ release.

**FIGURE 3:**
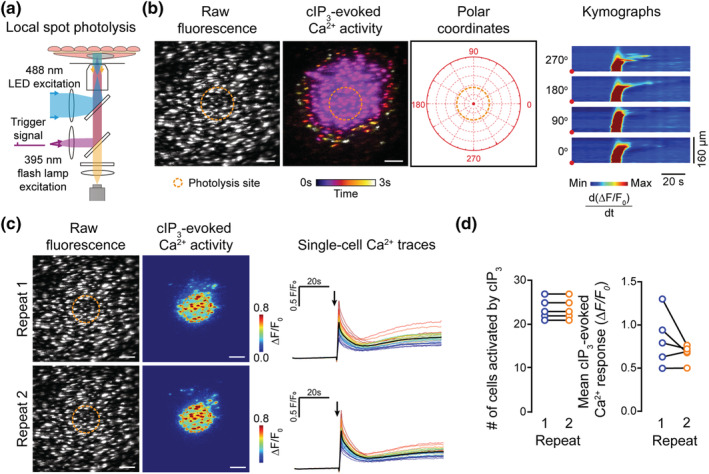
cIP_3_‐evoked increases in endothelial Ca^2+^ levels are reproducible. (a) Schematic of localised photolysis of cIP_3_ with simultaneous widefield endothelial cell imaging of intact arteries. (b) Representative Ca^2+^ images and kymograph illustrating temporal dynamics of cIP_3_‐evoked endothelial Ca^2+^ activity. Ca^2+^ images show raw fluorescence (left), temporally colour‐coded projection of active Ca^2+^ wave fronts (determined by sequential subtraction, middle; photolysis region shown by dotted line), and the polar coordinates used for the kymograph. (c) Example of raw and pseudocoloured Ca^2+^ images and corresponding single‐cell Ca^2+^ traces (black line average) illustrating the response of a single field of endothelial cells to repeat photolysis of cIP_3_ (30‐min equilibration between recordings). Arrow indicates uncaging event. (d) Summary data showing no significant differences in the number of cells activated by successive cIP_3_ photolysis events (left; 24 ± 1 cells for repeat 1, 24 ± 1 cells for repeat 2, *n* = 5) and the mean amplitude of the Ca^2+^ response (right; 0.80 ± 0.10 ΔF/F_0_ for repeat 1, 0.67 ± 0.05 ΔF/F_0_ for repeat 2; *n* = 5). All image scale bars = 50 μm

**FIGURE 4:**
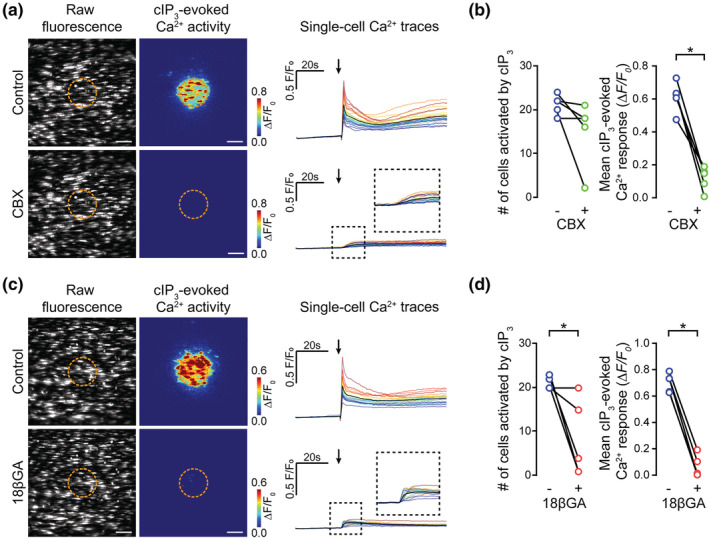
Putative gap junction blockers inhibit cIP_3_‐evoked endothelial Ca^2+^ responses. (a–d) Effect of carbenoxolone (a and b; CBX, 100 μM, 5 min incubation) and 18βGA (c and d; 40 μM, 45 min incubation) on cIP_3_‐evoked (5 μM) endothelial cell Ca^2+^ signalling. Panels (a) and (c) show raw baseline Ca^2+^ images, cIP_3_‐evoked Ca^2+^ activity images (pseudocoloured max ΔF/F_0_; dotted line shows photolysis site), and corresponding single‐cell Ca^2+^ traces (black line average) obtained from the same field of endothelial cells before and after incubation with the indicated inhibitor. Arrow indicates uncaging event. (b, d) Paired summary data plots showing the effect of the indicated inhibitor on the number of cells activated by cIP_3_ (left). For CBX, 21 ± 1 cells were activated in controls and 15 ± 3 cells after CBX (*n* = 5). For 18βGA, 21 ± 1 cells were activated in control and 8 ± 4 cells after 18βGA (*n* = 5). The mean amplitude of the cIP_3_‐evoked Ca^2+^ response (right) for CBX was 0.61 ± 0.04 ΔF/F_0_ in controls and 0.12 ± 0.03 ΔF/F_0_ after CBX (*n* = 5). For 18βGA, the mean amplitude of the cIP_3_‐evoked Ca^2+^ response was 0.70 ± 0.03 ΔF/F_0_ for controls and 0.06 ± 0.04 ΔF/F_0_ after 18βGA (*n* = 5). *P<0.05, significantly different as indicated; paired *t*‐test. All image scale bars = 50 μm

To determine if CBX and 18βGA interfere with the ability of IP_3_ to evoke Ca^2+^ release, the effects of the drugs on Ca^2+^ signals evoked by the photolysis of cIP_3_ were examined (Figures 3 and 4). Uncaged IP_3_ bypasses plasma membrane receptors to directly activate IP_3_ receptors. Photolysis of cIP_3_, in a 70 μm diameter spot, triggered an immediate rise in Ca^2+^ in the photolysis region followed by multicellular Ca^2+^ waves that propagated across cells away from the photolysis spot (Figure 3a,b, Video S2). The propagating waves encompassed the majority of the cells within the field of view (~330 μm width) by recruiting cells that had not been directly activated by photolysis of caged IP_3_ (Figure 3b; Video S2). The Ca^2+^ rise evoked by photolysis of cIP_3_ was reproducible on repeat activation. In cells within the photolysis spot (Figure 3c,d), there with no difference in either the number of cells activated by cIP_3_ or the amplitude of cIP_3_‐evoked responses in repeat activations.

CBX and 18βGA each decreased the extent of cIP_3_‐evoked Ca^2+^ wave propagation (Figure 4, Movies S3 and S4). However, CBX and 18βGA each also significantly inhibited Ca^2+^ activity evoked in cells directly activated by photolysis of cIP_3_ (i.e., Ca^2+^ activity in cells within the photolysis region, Figure 4; Movies S3 and S4). 18βGA and CBX decreased the amplitude of cIP_3_‐evoked responses in those cells directly activated by cIP_3_ photolysis (Figure 4b,d). 18βGA, but not CBX, also reduced the percentage of cells directly activated by cIP_3_ photolysis (Figure 4b,d). This result suggests that CBX and 18βGA each have a direct inhibitory action on IP_3_ receptors.

**FIGURE 5:**
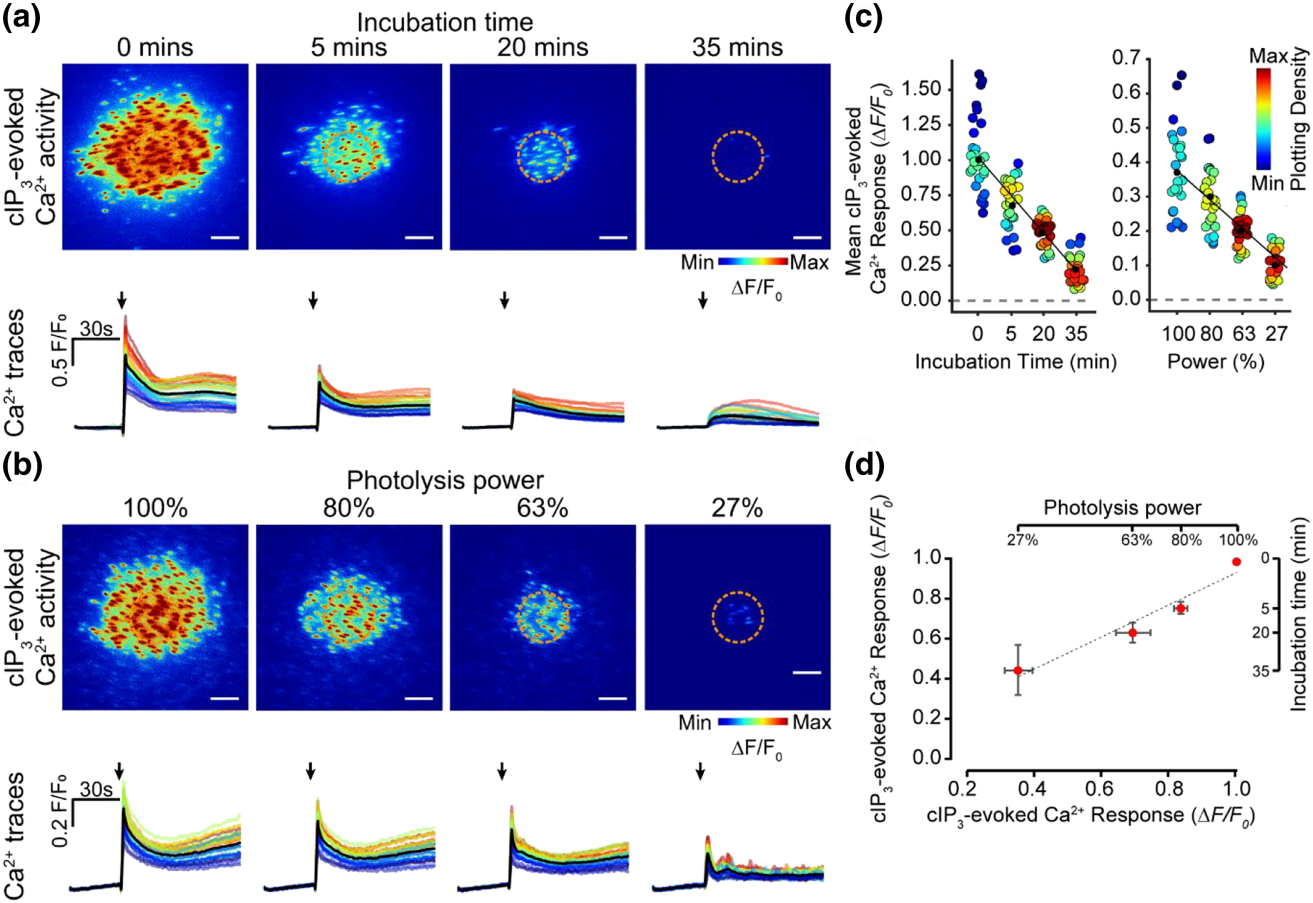
18βGA suppresses cell–cell communication by decreasing IP_3_‐evoked Ca^2+^ release. (a) Effect of 18βGA (40 μM) incubation for 5, 20, or 35 min on cIP_3_‐evoked endothelial cell Ca^2+^ signalling. (b) Effect of decreasing photolysis power on cIP_3_‐evoked endothelial cell Ca^2+^ signalling. Panels show cIP_3_‐evoked Ca^2+^ activity images (pseudocoloured max ΔF/F_0_ over 2 s post uncaging), and corresponding single‐cell Ca^2+^ traces (black line average) obtained from the same field of endothelial cells under the indicated condition. Arrows indicate uncaging event. (c) Mean cIP_3_‐evoked Ca^2+^ response from each cell shown in (a) (left) or (b) (right). Points are colour coordinated according to plotting density (blue low, red high), and a line of best fit plotted. (d) Scatterplot showing the relationship between cIP_3_‐evoked responses elicited at different stimulation intensities and those evoked after various 18βGA incubation times. Grey line shows the linear line of best fit (*n* = 5, error bars: SEM). Scale bars = 50 μm

To determine if CBX and 18βGA inhibition arose solely from a direct block of IP_3_‐evoked Ca^2+^ release, or if inhibition of gap junctions also contributed to the decreased Ca^2+^ response, we examined the time course of the 18βGA‐evoked reduction in cIP_3_‐evoked Ca^2+^ release. IP_3_‐evoked Ca^2+^ responses were recorded 10 min before 18βGA incubation (labelled 0 min; Figure 5a), and then at 15 min intervals (15 min were required for Ca^2+^ stores to replenish after photolysis of cIP_3_). This experimental protocol was not performed for CBX as the drug's inhibition of Ca^2+^ signalling was too rapid (<10 min). 18βGA evoked a time‐dependent reduction in (1) the amplitude of cIP_3_‐evoked Ca^2+^ signals within the photolysis site and (2) the outward propagation of Ca^2+^ signals from the photolysis site (Figure 5a,c. This result raises the possibility that the 18βGA‐mediated decrease in outward propagation of Ca^2+^ signals away from the photolysis site may arise from an inhibition of Ca^2+^ release rather than an inhibition of gap junction‐mediated communication.

To examine this possibility, we investigated the relationship between the extent of outward propagation of Ca^2+^ waves and the magnitude of initiating cIP_3_‐evoked Ca^2+^ release. The magnitude of Ca^2+^ release, initiated by the uncaging of cIP_3_, was scaled by the control of the photolysis light intensity. As the power of the photolysis light intensity was attenuated (using neutral density filters), there was a reduction in the amplitude of the Ca^2+^ response in the photolysis site and in the subsequent outward propagation of Ca^2+^ waves (Figure 5b). The relationships between the power of the photolysis stimuli and both the resulting amplitude of cIP_3_‐evoked Ca^2+^ response and the outward propagation of the Ca^2+^ signal were linear (Figure 5b,c). These results show that the initial Ca^2+^ signal amplitude and the outward propagation of the Ca^2+^ signal are proportional.

Significantly, the magnitude of initiating cIP_3_‐evoked Ca^2+^ release at various photolysis light transmission percentages plotted against magnitude of initiating cIP_3_‐evoked Ca^2+^ release occurring after increasing 18βGA incubation times shows a strong correlation (gradient of 0.82 and *R* ^2^ value of 0.95; Figure 5d). As the decrease in outward signal propagation was the same after intervention with either 18βGA (Figure 5b) or a decrease in photolysis light intensity (Figure 5a), this suggests that a major mechanism of action of the reported gap junction blockers is to inhibit IP_3_‐mediated Ca^2+^ release in the vascular endothelium of the mesenteric arteries.

**FIGURE 6:**
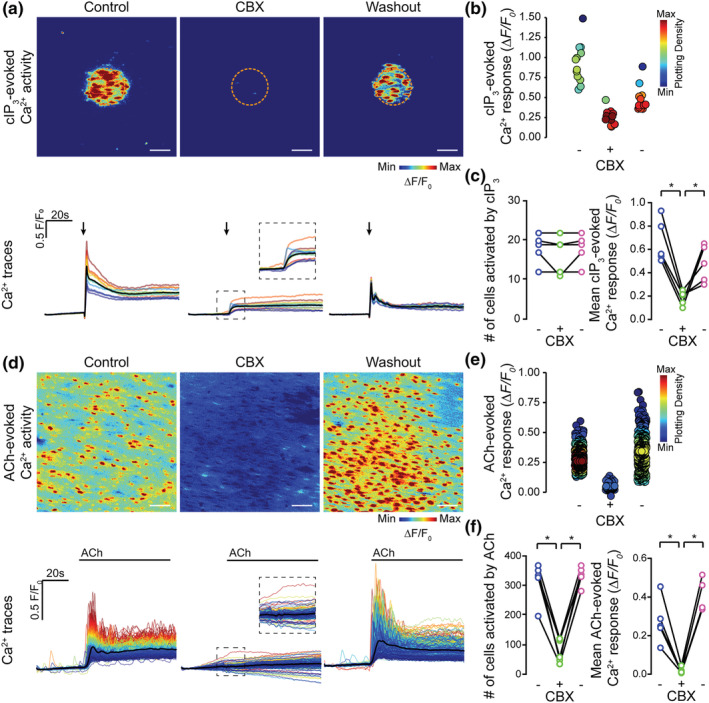
Inhibition of IP_3_‐mediated Ca^2+^ release by CBX is reversible. Effect of CBX incubation (100 μM, 5 min incubation) and washout (1 h, PSS) on (a–c) cIP_3_‐evoked (5 μM) and ACh‐evoked (d–f) endothelial cell Ca^2+^ signalling. Panels (a) and (d) show cIP_3_‐evoked Ca^2+^ activity images (pseudocoloured max ΔF/F_0_), and corresponding single‐cell Ca^2+^ traces (black line average) obtained from the same field of endothelial cells before and after incubation with, and after washout of, CBX. Arrows indicate uncaging event. (b, e) Mean Ca^2+^ response from each cell in the endothelial field shown under each condition. Points are colour coordinated according to plotting density; (c and f) paired summary data plots showing the effect of CBX incubation and washout on the number of cells activated (left) by cIP_3_ (c; 18 ± 2 cells in control vs. 17 ± 2 cells after CBX and 18 ± 2 cells after CBX washout; *n* = 5) and ACh (f; 315 ± 25 cells in control vs. 76 ± 18 cells after CBX and 332 ± 15 cells after CBX washout; *n* = 5). The mean amplitude of the Ca^2+^ response (c) for cIP_3_ was 0.67 ± 0.08 ΔF/F_0_ in control and 0.18 ± 0.03 ΔF/F_0_ after CBX, and 0.48 ± 0.07 ΔF/F_0_ following washout (*n* = 5). The mean amplitude of the Ca^2+^ response (f) for ACh was 0.27 ± 0.05 ΔF/F_0_ in control, 0.018 ± 0.007 ΔF/F_0_ in CBX and 0.50 ± 0.04 ΔF/F_0_ after CBX washout (*n* = 5). * *P*<0.05, significantly different as indicated; paired one‐way ANOVA with Tukey's multiple comparisons test. All image scale bars = 50 μm

**FIGURE 7:**
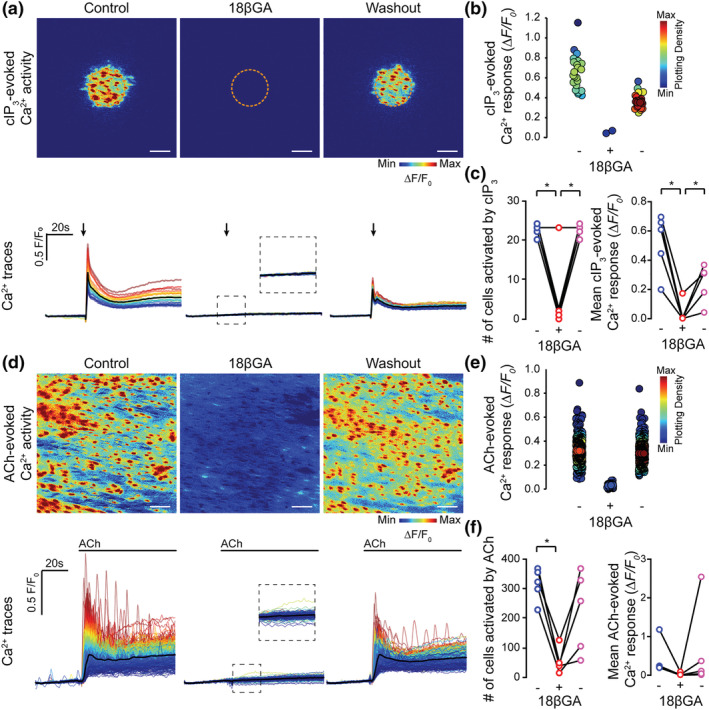
Inhibition of IP_3_‐mediated Ca^2+^ release by 18βGA is partly reversible. Effect of 18βGA incubation (40 μM, 45 min incubation) and washout (1 h, PSS) on (a–c) cIP_3_‐evoked (5 μM) and ACh‐evoked (d–f) endothelial cell Ca^2+^ signalling. Panels (a) and (d) show cIP_3_‐evoked Ca^2+^ activity images (pseudocoloured max ΔF/F_0_), and corresponding single‐cell Ca^2+^ traces (black line average) obtained from the same field of endothelial cells before and after incubation with, and after washout of, 18βGA. Arrows indicate uncaging event. (b, e) Mean Ca^2+^ response from each cell in the endothelial field shown under each condition. Points are colour coordinated according to plotting density; (c, f) paired summary data plots showing the effect of 18βGA incubation and washout on the number of cells (left) activated by (c) cIP_3_ (22 ± 1 cells in control, 6 ± 4 cells after 18βGA and 22 ± 1 cells after 18βGA washout; *n* = 5) and (f) ACh (317 ± 25 cells in control, 54 ± 19 cells after 18βGA and 225 ± 62 cells after 18βGA washout; *n* = 5). The mean amplitude of the Ca^2+^ response for cIP_3_ (c) was 0.52 ± 0.09 ΔF/F_0_ in controls, 0.04 ± 0.03 ΔF/F_0_ after 18βGA and 0.24 ± 0.06 ΔF/F_0_ following 18βGA washout (*n* = 5). For ACh (f), the mean amplitude of the Ca^2+^ response was 0.4 ± 0.2 ΔF/F_0_ in controls, 0.02 ± 0.02 ΔF/F_0_ after 18βGA and 0.6 ± 0.5 ΔF/F_0_ following 18βGA washout (*n* = 5). *P<0.05, significantly different as indicated; paired one way ANOVA with Tukey's multiple comparisons test. All image scale bars = 50 μm

CBX and 18βGA have each been reported to evoke cell death (Hasan et al., 2016; Lee et al., 2010; Yu et al., 2014). To investigate whether CBX and 18βGA decreased IP_3_‐evoked Ca^2+^ release by inducing cell death, the reversibility of the drugs was examined. IP_3_‐evoked Ca^2+^ signalling evoked by cIP_3_ or ACh was examined before incubation, after incubation, and after washout (1 h) of CBX (Figure 6) or 18βGA, (Figure 7). The inhibitory effects of CBX on Ca^2+^ release evoked by photolysis of IP_3_ (Figure 6a–c) or by ACh (Figure 6d–f) were reversed following drug washout. In these experiments, average Ca^2+^ ΔF/F_0_ responses significantly decreased during CBX and then significantly increased after the drug was washed out for both the cIP_3_‐evoked (Figure 6c) and ACh‐evoked (Figure 6f) activations. While the number of cells activated by cIP_3_ was unaltered by CBX (Figure 6c), the number activated by ACh was significantly decreased and reversed on washout (Figure 6f). Washout of 18βGA also resulted in a partial recovery of cIP_3_‐ and ACh‐evoked Ca^2+^ signalling (Figure 7). Although cIP_3_‐evoked Ca^2+^ responses were significantly decreased by incubation with 18βGA and ΔF/F_0_ increased again after washout (Figure 7c), the recovery was not significant for ACh‐evoked Ca^2+^ responses (Figure 7f). The number of cells was significantly decreased after incubation with 18βGA and increased again after washout for both IP_3_‐evoked signals (Figure 7c) and ACh‐evoked (Figure 7f). The greater recovery on washout for CBX is likely to be due to the increased water solubility of CBX, compared to 18βGA. These results suggest that CBX and 18βGA reversibly inhibit IP_3_‐mediated Ca^2+^ release.

To further test whether CBX and 18βGA caused cell death, we used propidium iodide staining as an assay of cell membrane permeability and apoptosis. Neither CBX nor 18βGA caused an increase in propidium iodide staining (Figure S1). Thus, in the present study, CBX and 18βGA did not evoke endothelial cell death, as measured by the reversibility of the IP_3_‐evoked Ca^2+^ responses and by propidium iodide staining.

**FIGURE 8:**
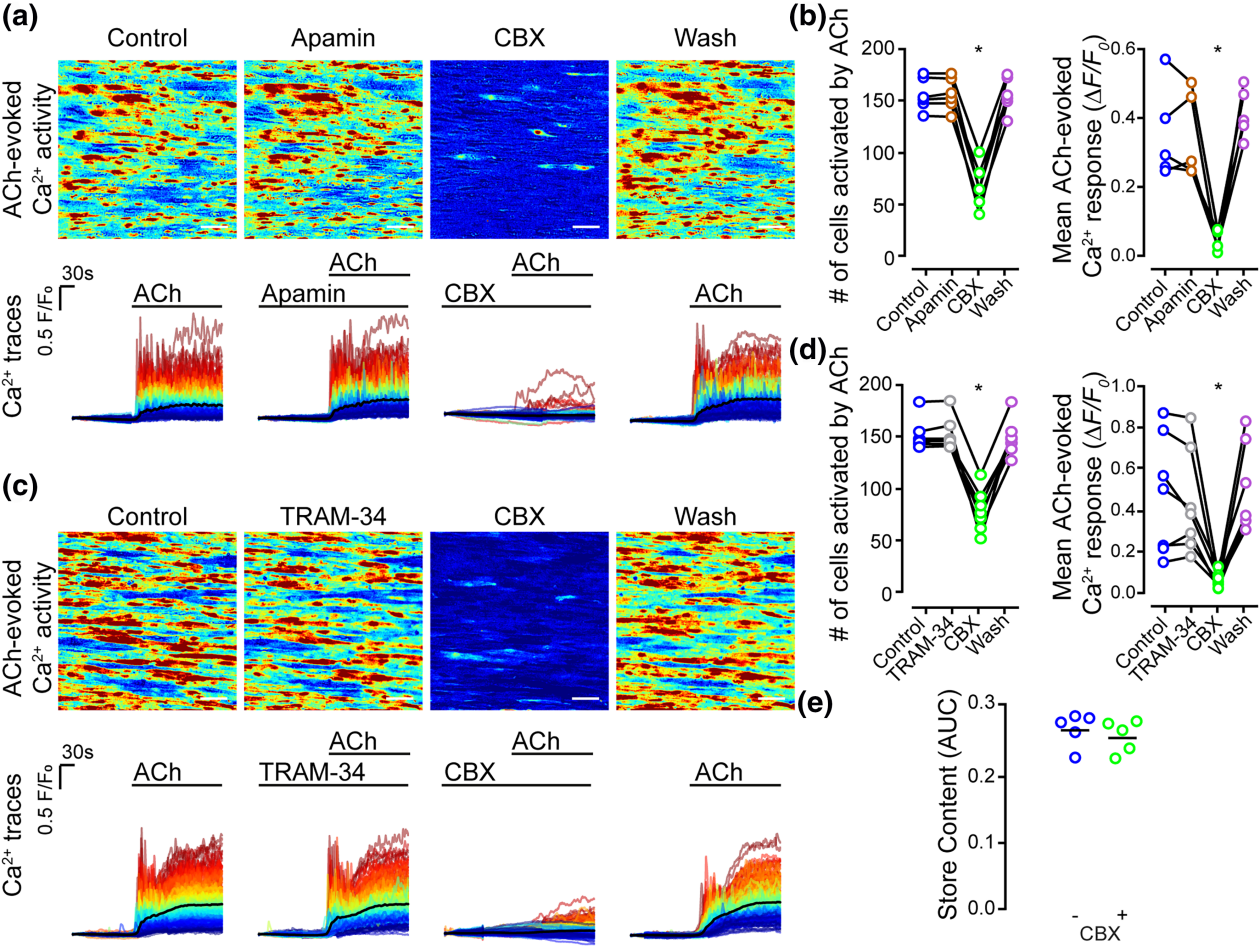
Inhibitory action of CBX is not due to blockade of small or intermediate conductance K^+^ channels nor is store content affected by CBX. Effect of (a, b) small (apamin, 100 nM, 10 min incubation) and (c, d) intermediate conductance (TRAM‐34, 1 μM, 10 min incubation) K^+^‐channel block on ACh‐evoked (100 nM) endothelial cell Ca^2+^ signalling. (e) Effect of CBX on store content, measured using CPA (5 μM in Ca^2+^ free PSS, 15 min). Panels (a) and (c) show ACh‐evoked Ca^2+^ activity images (pseudocoloured max ΔF/F_0_), and corresponding single‐cell Ca^2+^ traces (black line average) obtained from the same field of endothelial cells for a control recording, after incubation with TRAM‐34, after incubation with CBX, and after washout of all drugs; (b) paired summary data plots showing the effect of incubation of apamin and washout on the number of cells activated by ACh (left; 157 ± 6 cells in control, 157 ± 6 cells after apamin, 68 ± 9 cells in CBX and 157 ± 7 cells after washout, *n* = 6). The mean amplitude of the Ca^2+^ response (right) was 0.35 ± 0.06 ΔF/F_0_ in control, 0.35 ± 0.05 ΔF/F_0_ after apamin, 0.04 ± 0.01 ΔF/F_0_ after CBX and 0.41 ± 0.03 ΔF/F_0_ after washout (*n* = 5). (d) Paired summary data plots showing the effect of incubation of TRAM‐34 and washout on the number of cells activated by ACh (left; 151 ± 7 cells in control, 151 ± 7 cells after TRAM‐34, 83 ± 7 cells after CBX, 148 ± 8 cells after washout; *n* = 7) and the mean amplitude of the Ca^2+^ response (right) (0.5 ± 0.1 in control, 0.44 ± 0.09 ΔF/F_0_ after TRAM‐34,. 0.06 ± 0.02 ΔF/F_0_ after CBX and 0.50 ± 0.08 ΔF/F_0_ after washout ΔF/F_0_ ; *n* = 7). (e) Summary data showing the effect of CBX incubation on Ca^2+^ store content. *P<0.05, significant effect of CBX; paired one way ANOVA with Tukey's multiple comparisons test. All image scale bars = 50 μm

CBX and 18βGA are known to inhibit small (SK) and intermediate (IK) conductance K^+^ channels (Behringer et al., 2012) which may alter the plasma membrane potential and have consequences for Ca^2+^ store refilling (McCarron, Flynn, Bradley, & Muir, 2000). A block of store refilling could explain the effects of CBX and 18βGA on IP_3_‐evoked Ca^2+^ release. To determine if the inhibitory effects of CBX and 18βGA arose from K^+^‐channel‐dependent changes in membrane potential, IP_3_‐evoked endothelial Ca^2+^ responses were recorded in the absence and presence of apamin (100 nM, Figure 8a,b), an SK blocker, or TRAM‐34 (1 μM, Figure 8c,d), an IK blocker.

As shown in Figure 8a apamin did not alter ACh‐evoked Ca^2+^ signals, while CBX abolished the response in these same preparations (Figure 8a,b). Again, the effect of CBX was reversible on washout. The mean amplitude of ACh‐evoked Ca^2+^ signals and the number of ACh‐responsive cells (Figure 8b) confirms this.

TRAM‐34 also failed to alter ACh‐evoked endothelial Ca^2+^ signalling (Figure 8c,d). The mean amplitude of ACh‐evoked Ca^2+^ signals and number of ACh‐responsive cells (Figure 8d) were unaltered by the K^+^ channel blockers but were subsequently inhibited by CBX. As neither apamin nor TRAM‐43 altered IP_3_‐mediated Ca^2+^ release, it is unlikely that the inhibitory effects of CBX and 18βGA on IP_3_‐evoked Ca^2+^ release were mediated by K^+^ channel inhibition. The store content was unaltered in the absence and presence of CBX (100 μM, 5 min) as measured using the area under the whole field Ca^2+^ signal intensity curve upon addition of CPA (5 μM, 15 min) in a Ca^2+^‐free PSS (Figure 8e). The effectiveness of CPA‐induced store depletion was confirmed by the absence of a response to ACh (50 nM; not shown).

**FIGURE 9:**
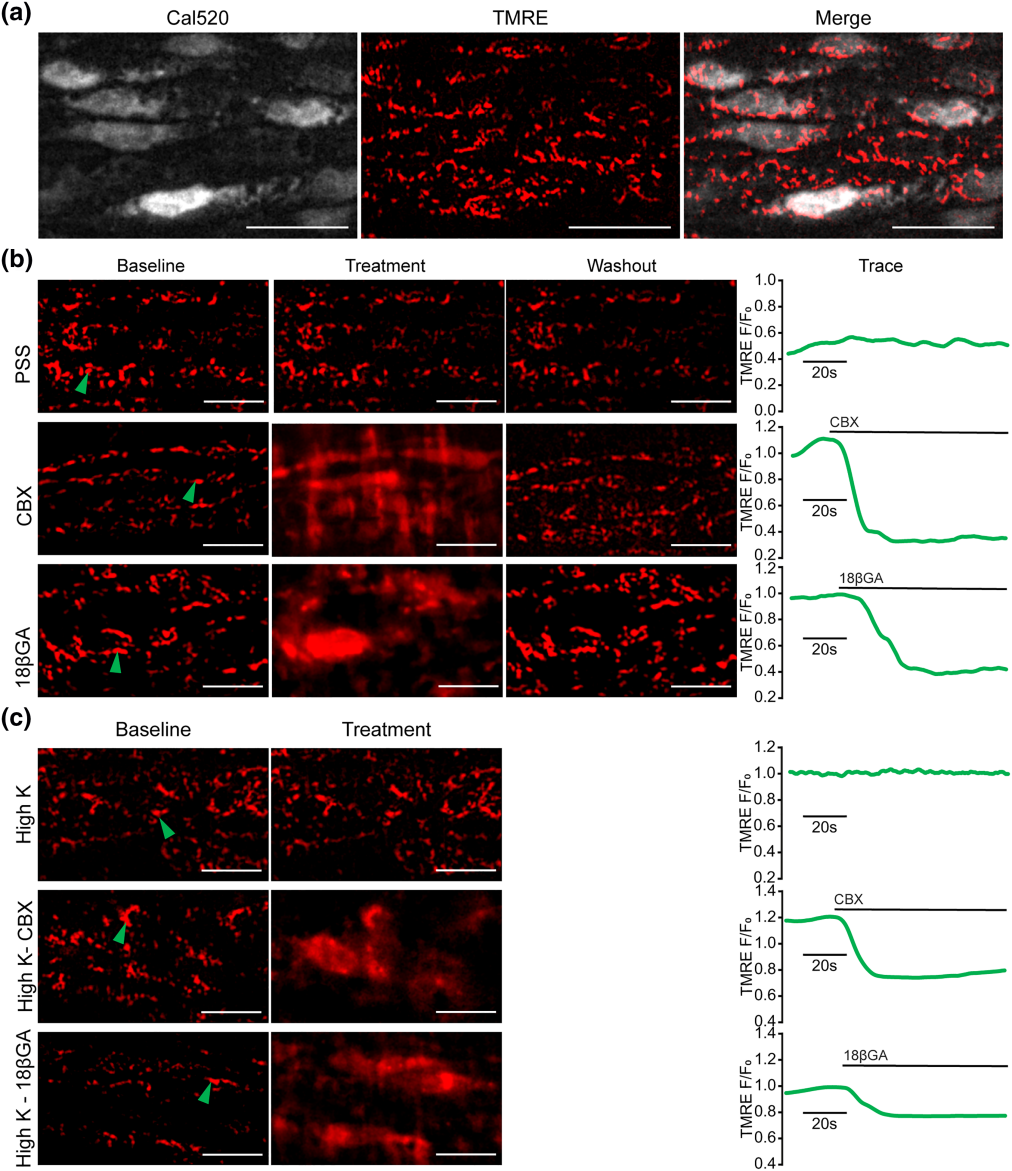
CBX and 18βGA each rapidly depolarise the mitochondrial membrane potential. (a) Endothelial cells from *en face* mesenteric artery preparations were stained with Cal‐520 (5 μM, grey) and TMRE (150 nM, red) to visualise the mitochondrial membrane potential (ΔΨ_M_). (b) Mitochondria were imaged for 1.5 min while administering PSS (control), CBX (100 μM), or 18βGA (40 μM) at 1.5 ml·min^−1^ under constant flow. Fluorescence intensity traces from individual mitochondria (designated by green arrows in the baseline image) are shown from across the treatment period for PSS, CBX, and 18βGA administration, indicated with by a bar over the trace. (c) Experiments were repeated in Ca^2+^‐free, high K^+^ PSS (control), CBX in Ca^2+^‐free, high K^+^ PSS and 18βGA in Ca^2+^‐free, high K^+^ PSS, and fluorescence intensity traces from individual mitochondria again shown. Examples from single experiments are shown from *n* = 5 biological replicates yielding similar results. Scale bars = 25 μm

CBX and 18βGA have been reported to collapse the mitochondrial membrane potential (ΔΨ_M_) (Salvi et al., 2005; Wang, Wong, Feng, & Zhang, 2014). Collapse of ΔΨ_M_ has widespread effects on cell function, including on the regulation of IP_3_‐evoked Ca^2+^ release (Alexander, Kelly et al., 2019; Correa et al., 2011; Csordas et al., 2006; Narayanan, Xi, Pfeffer, & Jaggar, 2010; Olson, Chalmers, & McCarron, 2010; Rizzuto, Brini, Murgia, & Pozzan, 1993; Rizzuto et al., 1998; Sward, Dreja, Lindqvist, Persson, & Hellstrand, 2002; Szado et al., 2003). To determine if the ΔΨ_M_ was altered by the drugs, mitochondria were visualised using the membrane potential indicator TMRE (120 nM, 5 min; Figure 9a) and the effects of CBX and 18βGA on ΔΨ_M_ were examined. CBX and 18βGA each evoked a rapid (within 60 s) and reversible depolarisation of ΔΨ_M_ (Figure 9b, baseline and treatment, Video S5), evident from the “smearing” of the punctate mitochondrial fluorescence signal as TMRE moves from the mitochondria into the cytoplasm. Equally striking was the speed at which mitochondria repolarised on washout of the drugs (Figure 9b, washout). Recovery occurred within 60 s of washout.

The concentration of TMRE in mitochondria is governed by Nernstian function of the mitochondrial membrane potential and plasma membrane potential. To ensure that the effect of CBX and 18βGA arose from depolarisation of ΔΨ_M_ rather than depolarisation of the plasma membrane potential, the plasma membrane potential was clamped using a high K^+^ PSS and the experiments repeated (Figure 9c). Ca^2+^ was omitted from the bathing solution to prevent smooth muscle contraction. In high K^+^‐PSS, CBX, or 18βGA each again rapidly depolarised ΔΨ_M_ (Figure 9c), as revealed by the loss of punctate TMRE staining. As the endothelial plasma membrane potential was clamped by the high K^+^‐PSS, the effect of CBX or 18βGA is on the mitochondria.

Taken together, these data suggest that CBX and 18βGA have pronounced effects on endothelial function by inhibiting IP_3_‐evoked Ca^2+^ release and depolarising ΔΨ_M_.

## 4 DISCUSSION

Ca^2+^ signals in the endothelium propagate regeneratively among cells to provide the long distance communication essential to coordinate normal vascular function (Lee et al., 2018; Longden et al., 2017; McCarron, Lee, & Wilson, 2017; Tallini et al., 2007; Wilson, Lee, & McCarron, 2016). Movement of small molecules such as IP_3_ or Ca^2+^ through gap junctions is proposed to underlie Ca^2+^ signal propagation and aberrant gap junction function may participate in cardiovascular disease development (Christ, Spray, el‐Sabban, Moore, & Brink, 1996; Pohl, 2020). The link between gap junctions and cardiovascular disease has generated a substantial interest in determining the contribution of gap junctions to cell communication. However, evaluation of the role of gap junctions has relied heavily on pharmacological interventions. Among the most frequently used pharmacological agents to assess the contribution of gap junctions to cell function are the blockers CBX and 18βGA. These pharmacological blockers are used often in intact tissue in which indirect measures of cell communication are employed. The present study presents experimental evidence that CBX and 18βGA are effective inhibitors of IP_3_‐mediated Ca^2+^ release and rapidly depolarise the mitochondrial membrane potential (ΔΨ_M_) when used at concentrations and incubation times reported to block gap junctions (Behringer et al., 2012; Boittin et al., 2013; Kim et al., 2017; Okamoto et al., 2014; Spray, Ye, & Ransom, 2006). Inhibition of IP_3_‐mediated Ca^2+^ release and ΔΨ_M_ depolarisation by CBX and 18βGA will result in widespread alterations in cell signalling and communication among cells but would not be discernible in indirect measures of cell communication. Careful consideration is therefore required in interpreting the results obtained from experiments in which CBX and 18βGA were used.

The mechanisms by which CBX and 18βGA block gap junctions are unclear (see Willebrords, Maes, Crespo Yanguas, & Vinken, 2017). 18βGA‐mediated inhibition of Cx43 may occur via dephosphorylation of type 1 or type 2A protein phosphatases (Guan et al., 1996), and direct interaction with the connexin has also been proposed to occur (Davidson & Baumgarten, 1988). There have been no studies clearly defining the mechanisms behind CBX inhibition of connexin channels (Leybaert et al., 2017). There are several reports of “off‐target” effects which may account for some of the effects of 18βGA and CBX on cell–cell communication. Glycyrrhetinic acids bind strongly to mineralocorticoid and glucocorticoid receptors (Armanini, Karbowiak, & Funder, 1983; Kratschmar et al., 2011), inhibit 11β‐hydroxysteroid dehydrogenase and act in anti‐inflammatory roles through these pathways (Morsy et al., 2019). CBX also shows high affinity for the mineralocorticoid receptor (Armanini, Karbowiak, Krozowski, Funder, & Adam, 1982).

In rat small mesenteric arteries, 18βGA (30 μM) blocked Ca^2+^ currents in smooth muscle cells (Matchkov, Rahman, Peng, Nilsson, & Aalkjaer, 2004). CBX (100 μM) also blocked voltage‐gated Ca^2+^ currents and reduced Ca^2+^ influx and depolarisation‐evoked Ca^2+^ signals in Salamander retina (Vessey et al., 2004). In cultured astrocytes, spontaneous action potentials, synaptic currents, and synchronised Ca^2+^ oscillations were also inhibited with 100 μM CBX, independently of gap junctions (Rouach, Segal, Koulakoff, Giaume, & Avignone, 2003). Cl^−^ currents were blocked by 40 μM 18βGA in primary rat hepatocytes (Bohmer, Kirschner, & Wehner, 2001) and delayed rectified K^+^ currents at concentrations up to 10 μM 18βGA in guinea pig myocytes. In endothelial tubes, IK/SK channel‐mediated hyperpolarisation was blocked by either 18βGA (up to 40 μM) or CBX (up to 100 μM) (Behringer et al., 2012). There are no previous reports of these drugs in the context of IP_3_ receptors or IP_3_‐mediated Ca^2+^ release, though other studies have found a reduction in IP_3_‐mediated activity upon CBX or 18βGA incubation but attributed the results to gap junction effects. For example, CBX (100 μM) inhibited ACh‐mediated Ca^2+^ release in the intact mouse aortic endothelium (Boittin et al., 2013) and blocked incremental IP_3_ increase in the guinea pig cochlea (Gossman & Zhao, 2008).

An alteration in K^+^‐channel activity (Behringer et al., 2012) by CBX and 18βGA could alter the plasma membrane potential and store refilling, providing an explanation for the decreased IP_3_‐evoked Ca^2+^ release. However, in the present study, there was no effect of either an SK‐channel blocker (apamin) or IK‐channel blocker (TRAM 34) on IP_3_‐mediated Ca^2+^ release. This suggests that inhibition of K^+^‐channel activity is an unlikely explanation of CBX‐ and 18βGA‐mediated inhibition of IP_3_‐mediated Ca^2+^ release in mesenteric artery endothelium.

Another unexpected finding in the present study was the rapid ΔΨ_M_ collapse induced by each of the gap junction blockers. The collapse of ΔΨ_M_ will have wide ranging effects on cell signalling. The CBX‐ and 18βGA‐induced collapse of ΔΨ_M_ (measured with TMRE) occurred in normal PSS and in a high K^+^ PSS that was used to clamp the plasma membrane potential. The concentration of TMRE in mitochondria is a Nernstian function of the ΔΨ_M_ and plasma membrane potential. Our finding that the change in TMRE fluorescence persisted in a high K^+^ PSS confirmed that CBX and 18βGA alter ΔΨ_M_, not the endothelial plasma membrane potential.

CBX and 18βGA have previously been reported to depolarise ΔΨ_M_. For example, in a pituitary adenoma cell line, 18βGA (up to 150 μM) caused a decrease in ΔΨ_M_ and elevated intracellular ROS and Ca^2+^ concentrations, stimulating mitochondrial permeability transition (MMP) leading to increased apoptosis (Wang et al., 2014). In ovarian carcinoma cell lines, 18βGA evoked apoptosis via potentiation of trichostatin A (1–25 μM, 24 h) (Lee et al., 2010) and ΔΨ_M_ depolarisation leading to Hsp90 inhibition‐mediated caspase 8 activation (Yang, Myung, Kim, & Lee, 2012) or cytochrome *c* release and caspase 3 activation (Lee, Kim, Lee, Han, & Lee, 2008). 18βGA‐induced mitochondrial membrane changes, and apoptosis occurs in human bladder cancer (Lin et al., 2011), human endometrial stromal (Yu et al., 2014), and human hepatoma cell lines (Hasan et al., 2016). CBX also induced ΔΨ_M_ collapse in liver mitochondria, resulting in mitochondrial permeability transition and apoptosis (Salvi et al., 2005).

While CBX and 18βGA each depolarised ΔΨ_M_, we did not observe endothelial cell apoptosis in the present study at the concentrations and incubation times used, as shown by the lack of propidium iodide‐positive staining (Figure S1) and the reversibility of the drug effects on Ca^2+^ signalling and ΔΨ_M_ depolarisation. CBX has better water solubility than 18βGA (Leybaert et al., 2017), and therefore, the washout of CBX was more effective than that of 18βGA. Notwithstanding, we did observe that leaving the drug on longer than the ~10 min for CBX or ~1 h for 18βGA caused a significant, irreversible increase in resting Ca^2+^ concentration in some cells (data not shown).

Collapse of ΔΨ_M_ by CBX and 18βGA could explain the changes in IP_3_‐evoked Ca^2+^ release (Alexander, Kelly et al*.,* 2019; Correa et al., 2011; Csordas et al., 2006; Narayanan et al., 2010; Olson et al., 2010; Rizzuto et al., 1993; Rizzuto et al., 1998; Sward et al., 2002; Szado et al., 2003). For example, in the endothelium, ROS such as hydrogen peroxide depolarise ΔΨ_M_ leading to inhibition of IP_3_‐evoked Ca^2+^ release (Alexander, Kelly et al*.,* 2019). The uncoupler carbonyl cyanide 3‐chlorophenylhydrazone (CCCP) or the complex 1 inhibitor, rotenone, also inhibited IP_3_‐evoked Ca^2+^ release in the endothelium by ΔΨ_M_ depolarisation (Alexander, Kelly et al., 2019). Our results therefore raised the possibility that CBX and 18βGA inhibit IP_3_‐mediated Ca^2+^ release by ΔΨ_M_ depolarisation. However, depolarisation of ΔΨ_M_ by CBX or 18βGA occurred rapidly (within 90 s) while inhibition of IP_3_‐mediated Ca^2+^ release developed more slowly (5 min for CBX; 45 min for 18βGA). The differences in time course suggests that ΔΨ_M_ depolarisation alone does not explain the inhibition of IP_3_‐evoked Ca^2+^ release and that CBX or 18βGA block IP_3_ receptors.

Together, our study questions the usefulness of CBX and 18βGA in studies on IP_3_‐mediated signal transduction via gap junctions in intact arterial tissue. CBX and 18βGA each inhibit IP_3_‐mediated Ca^2+^ release and depolarise ΔΨ_M_.

## AUTHOR CONTRIBUTIONS

C.B., C.W., and J.G.M. developed the concept. C.B. and X.Z. performed the experiments. C.B., X.Z., and C.W. analysed the data. C.B., X.Z., C.W., and J.G.M. interpreted the data. C.B. and J.G.M. drafted the manuscript. C.B., C.W., X.Z., and J.G.M. edited the manuscript. C.W., C.B., and J.G.M. sourced funding. All authors approved the final version of the manuscript.

## CONFLICT OF INTEREST

The authors declare no conflict of interest.

## DECLARATION OF TRANSPARENCY AND SCIENTIFIC RIGOUR

This Declaration acknowledges that this paper adheres to the principles for transparent reporting and scientific rigour of preclinical research as stated in the BJP guidelines for Design & Analysis, Immunoblotting and Immunochemistry, and Animal Experimentation, and as recommended by funding agencies, publishers and other organisations engaged with supporting research

## Supporting information


**Figure S1:** **Incubation with 18βGA and CBX does not increase membrane permeability**
(A,C) E*n face* mesenteric artery preparations were stained with Cal‐520 (5 μM, green) and propidium iodide (1.5 μM, red) and control recordings, recordings after incubation with (A) 18βGA (40 μM, 45 mins) or (C) CBX (100 μM, 5 mins), and after 1 hr washout (PSS, 1.5 ml.min^−1^). (B,D) The number of propidium iodide‐positive cells for each condition, plotted as a percentage of the total number of cells in the field of view. Examples from single replicates are shown from *n* = 5 paired biological replicates. Scale bars = 50 μm.

**Video S1: ACh evokes reproducible endothelial Ca** ^**2+**^ **increases**
E*n face* mesenteric artery preparation stained with Cal‐520 (5 μM, grey) was stimulated with ACh (50 nM) after 30s baseline recording (10 Hz). The resulting Ca^2+^ activity is visualised in green. Upper image: repeat 1; lower image: repeat 2, taken 30 mins after repeat 1. Scale bars = 50 μm.

**Video S2: cIP**_**3**_ **evokes reproducible endothelial Ca** ^**2+**^ **increases**
E*n face* mesenteric artery preparation stained with Cal‐520 (5 μM, grey) and cIP_3_ (5 μM). Ca^2+^ activity was stimulated by localised photolysis (yellow circle) after 30s baseline recording (10 Hz). The resulting Ca^2+^ activity is visualised in green. Upper image: repeat 1; lower image: repeat 2, taken 30 mins after repeat 1 in the same preparation. Scale bars = 50 μm.

**Video S3: CBX inhibits cIP**_**3**_ **‐evoked endothelial Ca** ^**2+**^ **responses**
E*n face* mesenteric artery preparation stained with Cal‐520 (5 μM, grey) and cIP_3_ (5 μM). Ca^2+^ activity was stimulated by localised photolysis (yellow circle) after 30s baseline recording (10 Hz). The resulting Ca^2+^ activity is visualised in green. A control recording was taken (upper image) after which the preparation was left to rest for 15 mins, then incubated in CBX (100 μM) for 5 mins. A second recording was then taken (lower panel). Scale bars = 50 μm.

**Video S4: 18βGA inhibits cIP**_**3**_ **‐evoked endothelial Ca** ^**2+**^ **responses**
E*n face* mesenteric artery preparation stained with Cal‐520 (5 μM, grey) and cIP_3_ (5 μM). Ca^2+^ activity was stimulated by localised photolysis (yellow circle) after 30s baseline recording (10 Hz). The resulting Ca^2+^ activity is visualised in green. A control recording was taken (upper image) after which the preparation was left to rest for 15 mins, then incubated in 18βGA (40 μM) for 45 mins. A second recording was then taken (lower panel). Scale bars = 50 μm.

Video S5: CBX rapidly depolarises the mitochondrial membrane potential
E*n face* mesenteric artery preparation were stained with TMRE (150 nM, grey) to visualise the mitochondrial membrane potential (ΔΨ_M_). Mitochondria were imaged for 1.5 mins whilst administering PSS only (upper panel) or PSS + CBX (100 μM) after a baseline recording of 30s at 1.5 ml.min^−1^ under constant flow. Scale bars = 25 μm.

## Data Availability

All data underpinning this study is available from the authors upon reasonable request.
