# Supplementary figures and images for: Carbenoxolone and 18β‐glycyrrhetinic acid inhibit inositol 1,4,5‐trisphosphate‐mediated endothelial cell calcium signalling and depolarise mitochondria
Source: Br J Pharmacol. 2021 Jan 17;178(4):896–912. doi: 10.1111/bph.15329 (PMC9328419; doi:10.1111/bph.15329)

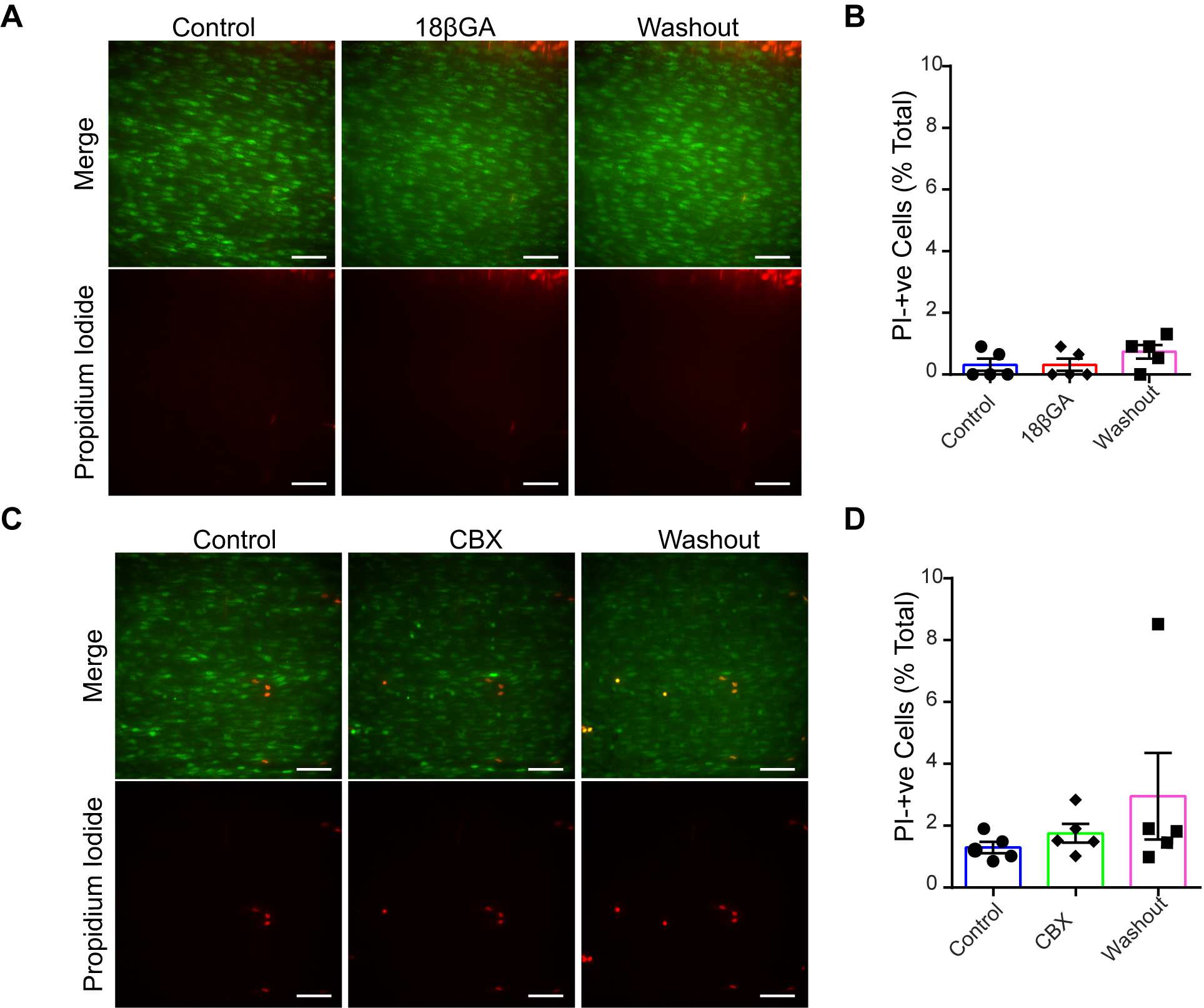

Supplement: Supplementary file 1 — Figure S1: Incubation with 18βGA and CBX does not increase membrane permeability (A,C) En face mesenteric artery preparations were stained with Cal‐520 (5 μM, green) and propidium iodide (1.5 μM, red) and control recordings, recordings after incubation with (A) 18βGA (40 μM, 45 mins) or (C) CBX (100 μM, 5 mins), and after 1 hr washout (PSS, 1.5 ml.min−1). (B,D) The number of propidium iodide‐positive cells for each condition, plotted as a percentage of the total number of cells in the field of view. Examples from single replicates are shown from n = 5 paired biological replicates. Scale bars = 50 μm. [file BPH-178-896-s001.tif]
